# The future of food allergy diagnosis

**DOI:** 10.3389/falgy.2024.1456585

**Published:** 2024-11-07

**Authors:** Dominic S. H. Wong, Alexandra F. Santos

**Affiliations:** ^1^ King's College London GKT School of Medical Education, London, United Kingdom; ^2^ Department of Women and Children’s Health (Paediatric Allergy), School of Life Course Sciences, Faculty of Life Sciences and Medicine, King’s College London, London, United Kingdom; ^3^ Peter Gorer Department of Immunobiology, School of Immunology and Microbial Sciences, King’s College London, London, United Kingdom; ^4^ Children's Allergy Service, Evelina London Children's Hospital, Guy's and St Thomas' Hospital, London, United Kingdom

**Keywords:** food allergy, diagnosis, molecular allergology, basophil activation test, mast cell activation test, bead-based epitope assay, T cell assays, microbiome analysis

## Abstract

Food allergy represents an increasing global health issue, significantly impacting society on a personal and on a systems-wide level. The gold standard for diagnosing food allergy, the oral food challenge, is time-consuming, expensive, and carries risks of allergic reactions, with unpredictable severity. There is, therefore, an urgent need for more accurate, scalable, predictive diagnostic techniques. In this review, we discuss possible future directions in the world of food allergy diagnosis. We start by describing the current clinical approach to food allergy diagnosis, highlighting novel diagnostic methods recommended for use in clinical practice, such as the basophil activation test and molecular allergology, and go on to discuss tests that require more research before they can be applied to routine clinical use, including the mast cell activation test and bead-based epitope assay. Finally, we consider exploratory approaches, such as IgE glycosylation, IgG4, T and B cell assays, microbiome analysis, and plasma cytokines. Artificial intelligence is assessed for potential integrated interpretation of panels of diagnostic tests. Overall, a framework is proposed suggesting how combining established and emerging technologies can effectively enhance the accuracy of food allergy diagnosis in the future.

## 1 Introduction

The prevalence of food allergy in Europe is increasing, with a recent meta-analysis showing a pooled lifetime prevalence of 19.9%, and a point prevalence of 13.1% for self-reported food allergy (1). US data from 2008 to 2016 show a 3.2-fold increase in emergency department visits connected to anaphylaxis (2). For those diagnosed, there are profound effects on every facet of their lives (3). Effects on mental health and productivity in the workplace are increasingly recognized, with wider implications for a healthy, cohesive society (4, 5). The development of accurate, economical tests for correctly diagnosing food allergy is essential to help combat these issues.

Through the next decade, recent findings and technologies hold immense potential to redefine the paradigm for food allergy diagnosis. Several novel modalities have been proposed to reduce the need for food challenges via non-invasive lab-based tests. These range across a variety of techniques, from conventional serology tests to artificial intelligence (AI) systems. In this narrative review, we will focus on IgE-mediated food allergy. First we will briefly describe the current status of food allergy diagnosis and summarize unmet needs in the field. We shall then discuss each novel approach in turn and explore future directions for food allergy diagnosis, delineating a pathway for the safe and effective integration of novel technologies into clinical practice, in the future. We have also generated Table 1 containing sensitivity and specificity data from selected studies across a range of modalities to provide a contextual background to the review.

**Table 1:** Sensitivity and specificity for food allergy diagnostic modalities from selected studies. BAT, basophil activation test; MA, molecular allergology; MAT, mast cell activation test; AI, artificial intelligence; DBPCFC, double-blind placebo-controlled food challenge; OFC, oral food challenge.

Author	Year	Technique	Food allergy	Tested allergen	Sample size	Cutoff/model	Reference test	Sensitivity	Specificity
Krawiec (11)	2023	BAT	Baked egg	Egg extract	150	%CD63 100 ng/ml	DBPCFC	78	77
Krawiec (11)	2023	BAT	Baked egg	Egg extract	150	SI CD203c 10 ng/ml	DBPCFC	78	78
Rubio (18)	2010	BAT	Cow's milk	Cow's milk extract	112	>6% CD63+	OFC/clinical history	91	90
Sato (76)	2010	BAT	Cow's milk	Cow's milk extract	71	SI CD203c≥1.9	OFC/clinical history	89	83
Sato	2010	BAT	Cow's milk	Casein	71	SI CD203c≥1.3	OFC/clinical history	67	71
Sato	2010	BAT	Cooked egg	Egg	71	SI CD203c≥2.4	OFC/clinical history	74	62
Sato	2010	BAT	Cooked egg	Ovomucoid	71	SI CD203c≥1.7	OFC/clinical history	80	73
Sato	2010	BAT	Raw egg	Ovomucoid	71	SI CD203c≥1.7	OFC/clinical history	77	63
Sato	2010	BAT	Raw egg	Egg white	71	SI CD203c≥1.6	OFC/clinical history	83	83
Ocmant (77)	2009	BAT	Cooked egg	Ovomucoid	67	≥5% CD63+	SPT	77	100
Ocmant	2009	BAT	Cooked egg	Ovomucoid	67	SI CD203c≥1.6	SPT	63	96
Tokuda (78)	2009	BAT	Wheat	Wheat	58	>11.1% CD203c+	OFC/clinical history	86	58
Tokuda	2009	BAT	Wheat	Omega-5 gliadin (nTri a 19)	58	>14.4% CD203c+	OFC/clinical history	86	58
Tokuda	2009	BAT	Wheat	Omega-5 gliadin (rTri a 19)	58	>7.9% CD203c+	OFC/clinical history	83	63
Santos (27)	2014	BAT	Peanut	Peanut	104	≥4.78% CD63+	OFC/clinical history	98	96
Glaumann (79)	2012	BAT	Peanut	Ara h 2	38	ND	DBPCFC	92	77
Brandstrom (80)	2015	BAT	Hazelnut	Hazelnut	40	CD-sens >1.7	DBPCFC	100	97
Alessandri (81)	2012	MA	Cow's milk	Bos d 4	66	>0 (kUa/l)	DBPCFC	56	88
Alessandri	2012	MA	Cow's milk	Bos d 4	66	>0.01 (kUa/l)	DBPCFC	62	88
Ando (82)	2008	MA	Egg	Gal d 1	108	>0.37 (kUa/l)	DBPCFC	97	36
Ando	2008	MA	Egg	Gal d 1	108	>4.4 (kUa/l)	DBPCFC	76	81
Ayuso (83)	2012	MA	Shrimp	rPen a 1	37	>0.35 kUa/l	DBPCFC	88	24
Klemans (84)	2014	MA	Peanut	Ara h 2	107	>0.3 ISU/l	DBPCFC	69	91
Klemans	2014	MA	Peanut	Ara h 2	107	>1.0 ISU/l	DBPCFC	59	95
Lieberman (85)	2013	MA	Peanut	Ara h 1	167	>0.35 kUa/l	DBPCFC	57	87
Lieberman	2013	MA	Peanut	Ara h 2	167	>0.35 kUa/l	DBPCFC	80	92
Masthoff (86)	2013	MA	Hazelnut	Cor a 1	161	>0.35 kUa/l	DBPCFC	80	7
Masthoff	2013	MA	Hazelnut	Cor a 8	161	>0.35 kUa/l	DBPCFC	6	96
Masthoff	2013	MA	Hazelnut	Cor a 9	161	>0.35 kUa/l	DBPCFC	60	88
Santos (26)	2018	MAT	Peanut	Peanut extract	174	17.2% of CD63+ LAD2 cells	OFC	73	98
Bahri (24)	2018	MAT	Peanut	Peanut extract	42	MAT-AUC 6.3	DBPCFC	97	92
Suarez-Farinas (29)	2021	BBEA	Peanut	Ara h 1, Ara h 2, Ara h 3	82	> 0.3 kUA/l	DBPCFC	91	92
Suarez-Farinas	2021	BBEA	Peanut	Ara h 1, Ara h 2, Ara h 3	84	> 0.3 kUA/l	DBPCFC	93	98
Kuniyoshi (69)	2020	AI		Combination	84	Logistic regression	OFC	70	73
Kuniyoshi	2020	AI	Cooked egg	Combination	84	Support vector machine	OFC	68	74
Kuniyoshi	2020	AI	Cooked egg	Combination	84	Extreme gradient boosting	OFC	51	66
Lin (87)	2012	AI	Peanut	Ara h 1, Ara h 2, Ara h 3	62	Decision tree	DBPCFC	87	94
Lin	2012	AI	Peanut	Ara h 1, Ara h 2, Ara h 3	62	Support vector machine	DBPCFC	90	97
Zhang (88)	2023	AI	Milk	α-Lactalbumin, β-Lactoglobulin, Cow Milk, Casein, Whey	1,112	Naïve Bayes ensemble	OFC	48	73
Zhang	2023	AI	Milk	α-Lactalbumin, β-Lactoglobulin, Cow Milk, Casein, Whey	1,112	Logistic regression ensemble	OFC	84	63
Zhang	2023	AI	Milk	α-Lactalbumin, β-Lactoglobulin, Cow Milk, Casein, Whey	1,112	Support vector machine ensemble	OFC	97	60
Zhang	2023	AI	Milk	α-Lactalbumin, β-Lactoglobulin, Cow Milk, Casein, Whey	1,112	Random forest ensemble	OFC	98	72
Zhang	2023	AI	Milk	α-Lactalbumin, β-Lactoglobulin, Cow Milk, Casein, Whey	1,112	Learning Using Concave and Convex Kernels ensemble	OFC	94	95
Gryak (89)	2024	AI	Peanut	Peanut, Ara h 1, Ara h 2, Ara h 3, Ara h 8 and Ara h 9	464	Learning Using Concave and Convex Kernels ensemble	OFC	99	100
Gryak	2024	AI	Peanut	Peanut, Ara h 1, Ara h 2, Ara h 3, Ara h 8 and Ara h 9	186	Naïve Bayes ensemble	OFC	76	98

## 2 Current clinical approach to food allergy diagnosis

Typically, food allergy is diagnosed through a combination of a thorough history and physical examination, and evidence of IgE to the suspected allergen, using skin prick testing and/or measurement of food-specific IgE in the serum (6). In patients with a clear history of an immediate reaction to a certain food and evidence of IgE to that food, the diagnosis of food allergy can be confirmed. Conversely, in individuals with no history of reacting to a food and no evidence of IgE sensitization to that food, the diagnosis of food allergy can be excluded. In all intermediate scenarios, additional tests need to be performed.

New guidelines from the European Academy of Allergy and Clinical Immunology (EAACI) (7) recommend initiating the diagnostic process with a detailed allergy-focused clinical history. Following this, skin prick test (SPT) and specific IgE (sIgE) to allergen extracts or the fresh food is strongly recommended. In some cases, these first-line tests do now allow a precise diagnosis, and for foods for which there are “informative components”, molecular allergology (MA) should be used. Examples of such informative components tests are sIgE to Ara h 2, Cor a 14, and Ana o 3, for peanut, hazelnut, and cashew allergies, respectively. For investigation of peanut or sesame seed allergies, basophil activation test (BAT) to peanut or sesame can be used, if available. BAT is a diagnostic technique involving basophil response which will be discussed in more detail in a later section. Of note, this was the first time molecular allergology and the BAT have been included in clinical guidelines to date.

Currently, the reference standard for precise assessment of food allergy is the Oral Food Challenge (OFC). Despite being expensive and carrying risks of anaphylaxis, OFC provides direct confirmation as to whether a patient will react to a given food allergen (8, 9). OFC procedures can also be tailored to answer specific clinical questions as needed. In equivocal cases, at various stages of the diagnostic process, OFC may be indicated and are ultimately the definitive diagnostic test currently. Open OFC are suitable for clinical practice and double-blind placebo-controlled food challenges are reserved for cases in which open OFC are, or have the risk of being inconclusive, and for research purposes.

The systematic review and meta-analysis conducted to inform the new EAACI food allergy clinical guidelines confirmed high sensitivity of SPT for the diagnosis of cow's milk and cooked egg allergy (90% and 94% respectively) (10). Sensitivity of sIgE to whole extract was 81%, 82%, 73/85%, and specificity 83%, 92%, 88/73%, for peanut, cow's milk, and raw/cooked egg respectively. In comparison to whole extracts, molecular allergology for individual components is highly specific for some foods. For instance, Ara h 2-sIgE has specificity of 92%, Cor a 14-sIgE 95%, Ana o 3-sIgE 94%, casein-sIgE 93%, ovomucoid-sIgE 91% for peanut, hazelnut, cashew, cow's milk, and cooked egg allergies, respectively.

**Figure 1:**
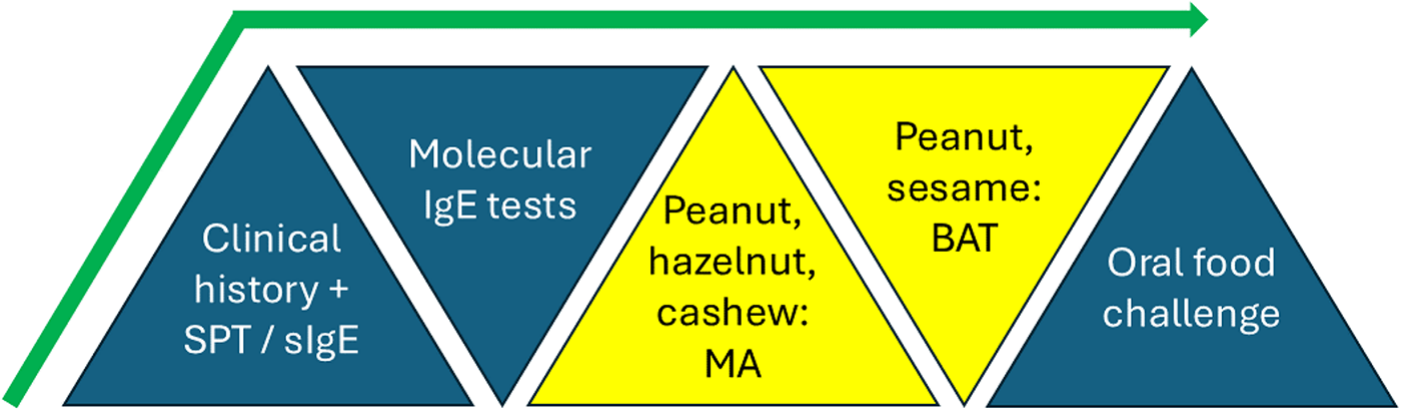
Recommended tests and their sequential use for diagnosis of food allergy in the new clinical guidelines for the diagnosis of IgE-mediated food allergy of the European academy of allergy and clinical immunology (EAACI). SPT, skin prick test; sIgE, specific IgE; MA, molecular allergology; BAT, basophil activation test.

The relatively lower sensitivity of components for milk and egg identified (67% and 74% for casein-sIgE and ovomucoid-sIgE respectively) were not superior to extracts, so were not included in the new guidelines. The BAT was both highly specific and highly sensitive for peanut and sesame, the only allergens for which there were sufficient numbers of BAT studies to perform meta-analyses (sensitivity and specificity of 84% and 90% for peanut, 89% and 93% for sesame). Since the review, BAT has also successfully displayed sensitivity and specificity of 78% and 77% to identify reactors to baked egg (11). Summary of the recommended tests in the new EAACI guidelines is shown in Figure 1.

## 3 Novel approaches in clinical practice

### 3.1 Molecular allergology

By characterizing molecular components of a given allergen that are recognized by patients' IgE, molecular allergology (MA) can be used to confirm suspected IgE-mediated food allergy (12). One of the main strengths of MA is that it allows for differentiation between co-sensitization and cross-reactivity, improving accuracy (13). The typical example is sensitization to plant foods secondary to pollen allergy—detecting IgE to the cross-reactive component (e.g., Ara h 8, Cor a 1) helps differentiate from primary systemic food allergy, associated with sensitisation to primary food allergens (for example, seed storage proteins Ara h 1/2/3/6 and Cor a 9/14). Further, MA has applicability to risk-stratification, helping to guide clinical management. Despite this, MA has demonstrated comparable specificity, but lower sensitivity, than OFC so far, limiting its value for primary diagnosis, with sensitivity of 67% and specificity 93% for casein, 74% and 91% for hen's egg ovomucoid, 82% and 92% for peanut Ara h 2 respectively (14). Exact values of sIgE titres are of limited clinical value, as severe reactions may still occur with sIgE of low levels and high affinity while conversely sIgE can also be present in the serum of resolved patients (15). Cost of MA is a limitation compared to SPT, though integration of novel multiplex platforms may help to minimize economic barriers, in the cases that require testing to many allergen components.

### 3.2 Basophil activation test

The BAT assesses degranulation of basophils *in vitro* following stimulation with the allergen. Basophils drive acute allergic responses via IgE recognition of the allergen and activation via the high-affinity IgE receptor. Following stimulation by allergens, flow cytometry is used to look for upregulation of the key markers CD63 and CD203c as a proxy for basophil degranulation. BAT has shown excellent diagnostic performance in clinical trials, having the potential to reduce the need for OFC (16). BAT has improved specificity compared to SPT and sIgE, while retaining sensitivity- limiting the number of false positives- with optimal cutoffs showing 98% sensitivity and 96% specificity for peanut (17). Further, BAT has potential utility for predicting disease trajectory, with some studies using repeated BAT to monitor acquisition of desensitization and remission of the allergic response (18). The ability of BAT to categorize allergic disease phenotypes through threshold dose reactivities offers opportunities for increased granularity of diagnostic approach, facilitating personalised care (19). Some of the barriers to further integration of BAT into clinical practice include the ca. 10% of individuals with basophils selectively unresponsive to FcεRI-mediated signalling. As an ex-vivo test on fresh (whole) blood, BAT features logistical difficulties in terms of procuring the sample from patients, requiring time-constrained transport to the lab and processing.

### 3.3 Low-dose and low-risk oral food challenges

Various approaches have been suggested attempting to improve feasibility and deliverability of OFC. In patients with minimal risk of reacting to the allergen, cumulative OFC has been adopted to reduce the time involved and the risk of desensitization during OFC, which has been associated with false-negative OFC. Cumulative OFC consists in a single dose OFC which resembles the age-appropriate portion size. Another approach often adopted for baked milk and baked egg is the low-dose OFC, which offers a way to establish a dose below which patients can continue eating the food at home. A lower dose has been used in a standard OFC, with one study defining the dose as 1%–10% of usual OFC dose (20, 21). The danger of a low dose OFC is the fact that the threshold of reactivity can change over time and allergen-content of foods is not always declared, thus care needs to be taken to avoid the allergen in circumstances known to be associated with a reduction in allergen threshold (e.g., infections, sleep deprivation, intense exercise, menstruation, etc), and to ingest foods with known allergen content that is within the recommended range for that patient.

## 4 Unmet needs

Primarily, there is a need for a non-invasive test that reduces the need for OFC to when absolutely necessary, due to the potential risk of a life-threatening anaphylactic reaction. Beyond risks to patients, logistically there is also limited capacity for OFCs due to its high resource-burden (22). It is noted that even in allergy centres with the highest capacity a prohibitively large imbalance between supply and demand results in long waiting lists. In the United Kingdom, contemporary data shows only 677 challenges per week over 139 centres (23). Reduced ability to deliver sufficient tests mandates prioritization of the most at-risk patients, and thence delay for much of the patient population. This carries a high cost, as delay in introducing foods back into the diet while immunoplasticity persists in young infants can increase the risk of developing food allergy later, especially in high-risk sensitized infants (9). Equally, late confirmation of food allergy being absent runs the risk of irreversible effects on patients’ food preferences and personal tastes.

There are also issues surrounding geographical equity; robust diagnosis of food allergy is currently limited to developed secondary and tertiary centres. This indicates an accessibility inequality in regions with limited care provision beyond primary care. Additionally, as therapies targeted to specific pathophysiological mechanisms are developed, accurately delineating the course of disease progression, and phenotypically stratifying diagnosed patients into discrete categories, will become increasingly more important.

## 5 Tests approaching clinical use

### 5.1 Mast cell activation test

Similarly to the BAT, the mast cell activation test (MAT) uses flow cytometry to observe the *in vitro* activation of mast cells to ascertain the degree of allergic response to particular allergens. Diagnostic performance of the MAT has been shown to exceed the SPT with an AUC of 0.99 compared to 0.73 for SPT (24). Mast cell lines or primary mast cells derived from peripheral blood hematopoietic stem cells are passively sensitized with the patient's serum or plasma, then incubated with allergen to assess response. The requirement for serum/plasma rather than fresh blood is a key distinguishing factor between BAT and MAT, potentially improving the feasibility of MAT and allowing for testing samples collected prospectively over time in the same experiment. However, growing mast cells can be challenging and primary cells are difficult to isolate, require tissue or large volumes of blood and are highly variable, with passive sensitization limiting sensitivity significantly (25). Requiring transfer of IgE to cells that are not autologous, MAT has lower sensitivity than the BAT with one study showing 75% to peanut compared to over 83% for BAT, so could be more useful in a sequential process following BAT, especially in cases of basophil-non-responders (26, 27).

### 5.2 Bead-based epitope assays

The bead-based epitope assay (BBEA) represents a scalable, high through-put multiplex assay for IgE and IgG4 to several sequential epitopes, requiring microliter samples only (28). This technology utilises peptides mechanically coupled to beads, which are incubated with serum or plasma samples, followed by quantification of fluorescence by fluorophore-labelled antibodies. Sensitivity and specificity of epitope detection, at 92% and 94% respectively, has been defined for peanut allergy. BBEA can also be used for phenotypic stratification and severity prediction (29, 30). A particular strength of BBEA is its reproducibility, which is key for effective widespread use. Errors pertaining to technical details (well position, reading order), as well as batch effects, are possible limitations.

BBEA requires precise prior knowledge of epitope amino acid sequences corresponding to each allergenic food of interest (29). Further implementation of BBEA is therefore predicated on continued advances in basic research concerning protein sequencing, as well as the sharing and processing of large epitope datasets developed from robustly characterised patient cohorts. Consequently, in the near future, BBEA likely has most applicability to diagnosis of the well-researched peanut allergy. An important limitation is that BBEA is limited to sequential epitopes, whereas in reality conformational epitopes also play a key role in food allergy. BBEA analysis also does not capture the totality of the IgE response, as it does not reflect components such as affinity and cellular response which are better characterized by the cellular assays mentioned earlier, as well as only demonstrating allergen sensitisation rather than overt clinical allergy (31). Utility of BBEA results may therefore be improved with parallel investigation of cellular assays, and correlation with clinical history.

## 6 Exploratory approaches

### 6.1 Glycosylation of allergen and glycosylation of IgE

Glycosylation of natural allergens can lead to cross-reactivity mediated by glycan-specific antibodies (14, 32). For instance, sensitization to cross-reactive carbohydrate determinants (CCD) can cause detectable IgE to peanut whilst resulting in negative SPT or BAT to peanut, reflecting the absence of clinical relevance in this CCD sensitisation. In Ghanaian children, CCD was identified as the underlying reason for IgE cross-reactivity between IgE to peanut and IgE to parasites, such as Schistosoma Mansoni (33). While IgE to glycans is often regarded as a barrier to specificity of *in vitro* diagnostics, it could represent another component of the allergic response to fully investigate in addition to conventional IgE serology, defining distinct patient sensitization profiles (34). For instance, IgE to α-Gal has previously been associated with meat-allergy delayed anaphylaxis (35).

A different prism is that of IgE glycosylation, which has been implicated in the pathogenicity of IgE in its ability to induce mast cell and basophil degranulation *in vitro*, and anaphylaxis in a mouse model, when compared with non-allergic individuals (36). However, the functional consequences of differences in IgE glycosylation between allergic and tolerant individuals has yet to be assessed between allergen-specific IgE molecules.

### 6.2 Allergen-specific IgG4 and IgA

Some studies have shown potential significance in testing for specific IgG4 against foods (37, 38). IgG4 is an IgG subclass that is produced under the influence of the same cytokines as IgE and has the unique ability for monovalent bispecific binding via Fab-arm exchange, theorized to be associated with IgE class-switching. However, it has been suggested that IgG4 merely represents a bystander of immunological tolerance rather than a factor driving such immune response (39). In the LEAP study, peanut-specific IgG4 increased over time in the study arm who consumed peanut from the first year of life, who also had less peanut allergy (40). In a recent cross-sectional egg allergy study, sIgG4 to egg, egg white and egg components, ovomucoid and ovalbumin, was higher in allergic compared with tolerant children and in consumers compared with avoiders (41). More research is needed to understand the role of IgG4 in food allergy and, certainly, to understand its utility in food allergy diagnosis.

sIgA is also presumed to play a role in the pathophysiology of food allergy, as sIgA deficiency has been linked to higher rates of allergies including food allergy, and salivary IgA has correlated with oral immunotherapy response for peanut allergy (42, 43). One cohort study indeed revealed higher plasma sIgA counts in children with peanut allergy, though no evidence was found for protection of food-specific IgA against development of food allergy or its potential utility for food allergy diagnosis (44).

### 6.3 T cell assays

A novel food allergy biomarker involves gauging the frequency of pathogenic T cell subsets and their activation in response to allergen stimulation. T cells are key immune cells in orchestrating food allergic and tolerant responses. In particular, Th2A cells, a subset of terminally differentiated memory CD4 + helper T cells, with characteristic expression of CD molecules and distinct functionality from conventional Th2 cells, have been associated with a specific stable allergic disease-related phenotype (45). One study examining pathophysiological trajectories of peanut allergy in a cohort of patients receiving oral immunotherapy showed clinical utility of subgroup division according to peanut-specific T-cell mediated immune profile (46). Discrete peanut-reactive T cell immunotypes based on relative proportions of Th2A cells were demonstrated, which were significantly related to distinct responses to allergen-specific immunotherapy. Additionally, Tfh13 cells, a subset of T follicular helper cells, may also be implicated in inducing anaphylaxis through induction of high-affinity specific IgE via sequential switching of B cells from IgG1 to IgE (47). In a study elucidating the position of T regulatory cells in spontaneous cow's milk allergy recovery, artificial neural networks were used to identify the markers FoxP3, Nfat-C2, IL-16, and GATA-3 as associated with the persistence of cow's milk allergy, with higher FoxP3 intensity in cow's milk allergic children, linking Th2 and Treg expression profiles with food allergy disease activity (48). An enhanced understanding of further Th subtypes could permit personalised care via assessment of individual T cell phenotypic signatures, designing and delivery of novel immunomodulatory therapies, as well as prediction of treatment response.

### 6.4 B cell assays

Playing an integral role in the production of allergen-specific antibodies, characterization of B cells is another possible biomarker for food allergy, determining specific attributes of the B cell receptor. IgE + B cells are directly related to the pathogenesis of food allergy, but are low in frequency and therefore difficult to identify. Investigative studies into IgE + B cells have identified the majority of IgE + B cells as immature plasmablasts with a common clonal family (49). IgE + memory B cells have been detected in the circulation, but they can also be present in the tissue, namely in the stomach and duodenum (50, 51). IgE + clonal signatures from gut samples could therefore represent one potential avenue to identify food allergy, though limited by access to relevant tissue sites in the gut. As with T cells, specific B cell subsets may also allow for further insights into food allergy, such as type 2-polarised memory B cells which are implicated in generating sIgE in early sublingual immunotherapy for birch pollen/house dust mite allergy (52). Similarly, a unique population of CD23 + IgG1 + memory B cells has also been described in the long-term persistence of peanut allergy in children, with frequency correlating to serum IgE levels (53, 54).

### 6.5 Microbiome analysis

In a similar vein to characterization of T cell responses, studying microbiome dynamics could permit discrete categorisation of food allergy trajectories. Disparities in the prevalence of certain bacterial species between peanut-allergic and non-allergic patients were demonstrated in a multidimensional multi-omic study of the oral microbiome, accompanied by significant differences in short-chain fatty acid and IL-4 titres (55). In particular, abundances of Prevotella species were protective against a Th2-mediated response, whereas milieus tending towards Neisseria categorized Th2-mediated allergic response. This corroborates with literature suggesting a role for Neisseria-related mucosal inflammation. In a similar study using faecal samples, a metabolome network analysis from infancy to mid-childhood was conducted, revealing significant associations between reduced microbiome diversity, temporal bacterial variation, and histidine, butyrate, isovalerate pathway metabolites with development of peanut allergy (56).

Iterating on the evidence provided by associative trials, oral and gut microbiomes have been clinically implicated in influencing disease reaction thresholds. A prospective multiscale study revealed characteristic oral and gut microbiome signatures affiliated with peanut allergy, observing significant associations between prevalence of *Veillonella* and *Bacteroides* species with high-threshold peanut-allergic patients (57). Consistency of ordinal trends was also noted between these discriminative bacteria and non-allergic participants. These findings suggest integrated analysis of combined oral and gut environments could be worthwhile in exploring crosstalk and categorical summation of host-microbe-immune dynamics. Advances in microbiome analysis may be laterally transferable to other allergenic foods, as pro-allergenic micro-environments are likely to be shared between food allergies without requiring precise knowledge of peptide sequences, as in BBEA. Despite modest predictive performance in preliminary research, the complex and interdependent mechanisms of the gut and oral microbiome create fundamental limitations for the specificity of any proposed diagnostic test involving the microbiome, as confounding effects (diet, ethnicity, comorbidities) may create statistical noise, making microbiome analysis unlikely to be significantly useful outside the context of a broader picture of host metabolites and IgE levels (58, 59).

### 6.6 Plasma cytokine analysis

As a natural extension from effector immune cell responses, characterization of the cytokine milieu could further define patient-specific immunopathogenetic signatures. Plasma cytokine profiles have been studied as predictors of food allergy diagnosis and resolution among sensitized infants, with specific reference to higher levels of allergy-defining IL-4, IL-13, IL-12p70, and lowered IL-10 (60, 61). Heterogeneity of cytokine profile has also been described between peanut and egg-allergic patients, which has been related to disparities in resolution rate (62). One prospective study explored the clinical utility of these markers, observing significantly raised median TNF- α and IL-6 levels before a therapeutic elimination diet in food-allergic participants, followed by significant decreases post-diet. The existing literature suggests a role for plasma cytokines in quantifying success of immunotherapeutic interventions, with decreases in IL-5 and IL-13 observed after food allergen immunotherapy, though it is worth noting the transience of this immunologic suppression (63, 64). These results are positive toward integration of plasma cytokine levels in initially confirming food allergy and tracking disease trajectory, but must be interpreted with particular consideration to time-course due to their fluctuant nature. More utility may therefore be derived from multiple readings longitudinally and single-cell cytokine studies. The efficacy of single-cell cytokine analysis is highlighted in the work of Chiang et al., who used single-cell profiling to interrogate heterogenous Th2 effector subsets in peanut-allergic patients, revealing a delayed, IL-2-dependent CD154 upregulation on Treg cell marker-expressing cells (65). Similarly, Neeland et al. showed significantly greater Il-6 and TNFα production, and lower IFN-γ production, with single-cell analysis of CD3/CD28 stimulated naïve CD4+ T cells in food allergic adolescents (66).

### 6.7 Artificial intelligence

AI methods hold a revolutionary potential for precisely identifying food allergy and synthesizing results of various tests for enhanced diagnostic accuracy. In comparison to conventional computerised protocols, artificial intelligence is able to fluidly adapt to provided datasets; learning from prior experience and applying this to novel situations. Specifically, machine learning (ML) frameworks, defined as a subset of AI involving teaching machines to emulate human learning while avoiding explicit programming, have utilized biomarkers including omics, total IgE, sIgE, sIgG4, and SPT data to successfully predict food allergy in infant populations (67). ML has also been efficacious as part of a medical algorithm identifying food allergy from data on methylation of highly discriminating cytosine-phosphate-guanine dinucleotides, reaching 100% accuracy through an ensemble voting scheme (68). Furthermore, prediction of tolerance to oral food challenge has been successfully demonstrated with Random Forest model sensitivity and specificity of 98% and 95% respectively for heated egg (69). ML processes have also been integrated within the novel bead-based epitope profiling mechanism described earlier, with the ability to accurately examine baseline variables influencing response to oral immunotherapy (70).

One of the major benefits of artificial intelligence approaches is the possibility for exploratory, unbiased data-driven analysis such as K-Means Clustering, which is an unsupervised algorithm enabling the grouping of unlabelled datasets into discontinuous clusters. Effective application of this to the MAT technique has been demonstrated, creating distinct clusters of phenotypes within a dataset of patients with similar responses to MAT (24, 71). AI models have also been successfully applied to MA multiplex arrays (72). As described in previous sections, the process of food allergy diagnosis lends itself to categorization of specific phenotypes in continuous unlabelled datasets, indicating high potential to accelerate our understanding of allergic patient presentations across a wide array of allergens and laboratory techniques. As well as this lateral applicability, there is also immense forward capability for analysis of methods that have yet to be discovered or refined, permitting swifter validation and optimization.

## 7 Conclusion

It is worth noting that no one modality described in this review is likely to be the perfect discriminant of food allergy. Rather, combinations of tests have been postulated to maximize diagnostic accuracy (73, 74). In the short-term, upscaling of existing, proven technologies such as MA and BAT, that have recently begun to be introduced into clinical practice may carry the timeliest return-on-investment in achieving tangible improvements in quality-of-care for patients with suspected food allergy. Laboratory tests made more streamlined, high-throughput, and affordable will be impactful in improving access to food allergy diagnosis in under-resourced communities. This requires a parallel investment in laboratory processing capabilities, and development of rapid, efficient processes on a macroscopic systems level.

**Figure 2:**
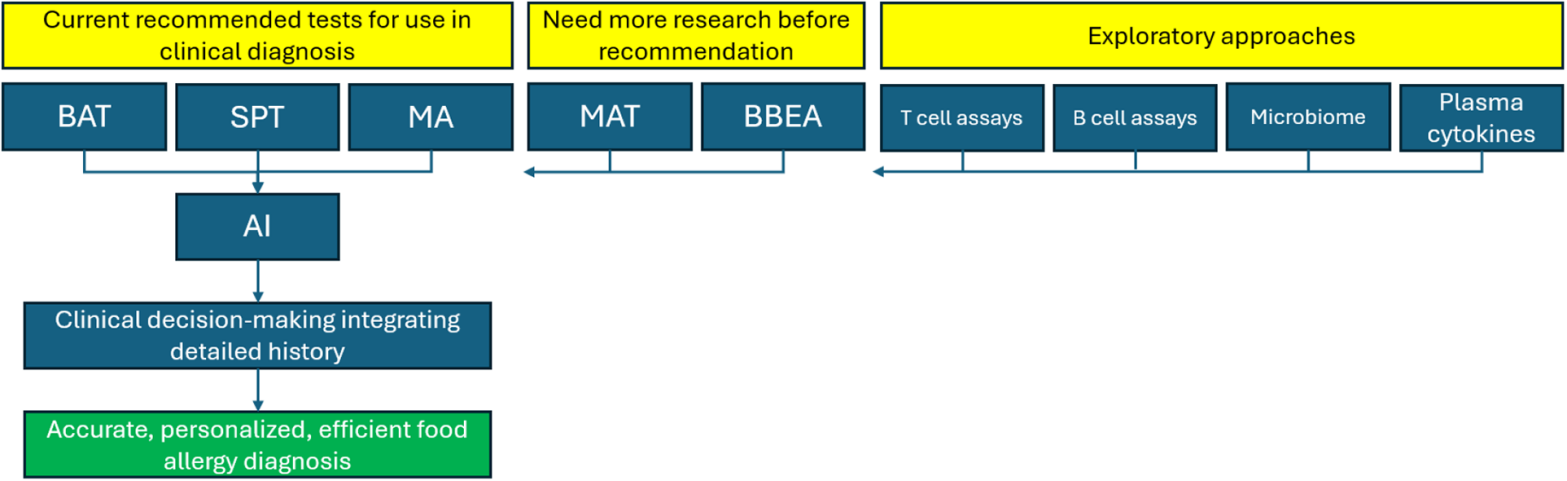
Framework for future directions in the diagnosis of IgE-mediated food allergy. BAT, basophil activation test; SPT, skin prick test; MA, molecular allergology; MAT, mast cell activation test; BBEA, bead-based epitope assay; AI, artificial intelligence.

Alongside this, ongoing research into the exploratory techniques described in this review will pay dividends for the treatment and management of food allergy on a long-term scale. As advances are made in targeted immunotherapy, increased depth and granularity of food allergy diagnosis will be necessitated to access its full potential. For example, personalized biologic treatments could be selected based on a patient's unique signature of cellular response, cytokine milieu, and microbiome. Underlying this, a data-driven approach guided by high-quality trained ML networks will be essential for enhanced interpretation of intricate omics datasets (75). A suggested pathway for direction of future food allergy diagnostics is shown in Figure 2.

Overall, this review suggests the rapidly changing face of food allergy diagnosis is well-positioned to address the projected increase in clinical burden in the near future, through an integrated approach combining laboratory technologies, artificial intelligence, and the art of medicine, to make a precise and timely diagnosis for every single food allergy in each individual.
